# Prevention of HPV-Related Cancers in Norway: Cost-Effectiveness of Expanding the HPV Vaccination Program to Include Pre-Adolescent Boys

**DOI:** 10.1371/journal.pone.0089974

**Published:** 2014-03-20

**Authors:** Emily A. Burger, Stephen Sy, Mari Nygård, Ivar S. Kristiansen, Jane J. Kim

**Affiliations:** 1 University of Oslo, Department of Health Management and Health Economics, Oslo, Norway; 2 Harvard School of Public Health, Department of Health Policy and Management, Center for Health Decision Science Boston, Massachusetts, United States of America; 3 Cancer Registry of Norway Department of Research, Oslo, Norway; Georgetown University, United States of America

## Abstract

**Background:**

Increasingly, countries have introduced female vaccination against human papillomavirus (HPV), causally linked to several cancers and genital warts, but few have recommended vaccination of boys. Declining vaccine prices and strong evidence of vaccine impact on reducing HPV-related conditions in both women and men prompt countries to reevaluate whether HPV vaccination of boys is warranted.

**Methods:**

A previously-published dynamic model of HPV transmission was empirically calibrated to Norway. Reductions in the incidence of HPV, including both direct and indirect benefits, were applied to a natural history model of cervical cancer, and to incidence-based models for other non-cervical HPV-related diseases. We calculated the health outcomes and costs of the different HPV-related conditions under a gender-neutral vaccination program compared to a female-only program.

**Results:**

Vaccine price had a decisive impact on results. For example, assuming 71% coverage, high vaccine efficacy and a reasonable vaccine tender price of $75 per dose, we found vaccinating both girls and boys fell below a commonly cited cost-effectiveness threshold in Norway ($83,000/quality-adjusted life year (QALY) gained) when including vaccine benefit for all HPV-related diseases. However, at the current market price, including boys would not be considered ‘good value for money.’ For settings with a lower cost-effectiveness threshold ($30,000/QALY), it would not be considered cost-effective to expand the current program to include boys, unless the vaccine price was less than $36/dose. Increasing vaccination coverage to 90% among girls was more effective and less costly than the benefits achieved by vaccinating both genders with 71% coverage.

**Conclusions:**

At the anticipated tender price, expanding the HPV vaccination program to boys may be cost-effective and may warrant a change in the current female-only vaccination policy in Norway. However, increasing coverage in girls is uniformly more effective and cost-effective than expanding vaccination coverage to boys and should be considered a priority.

## Introduction

**Figure 1:**
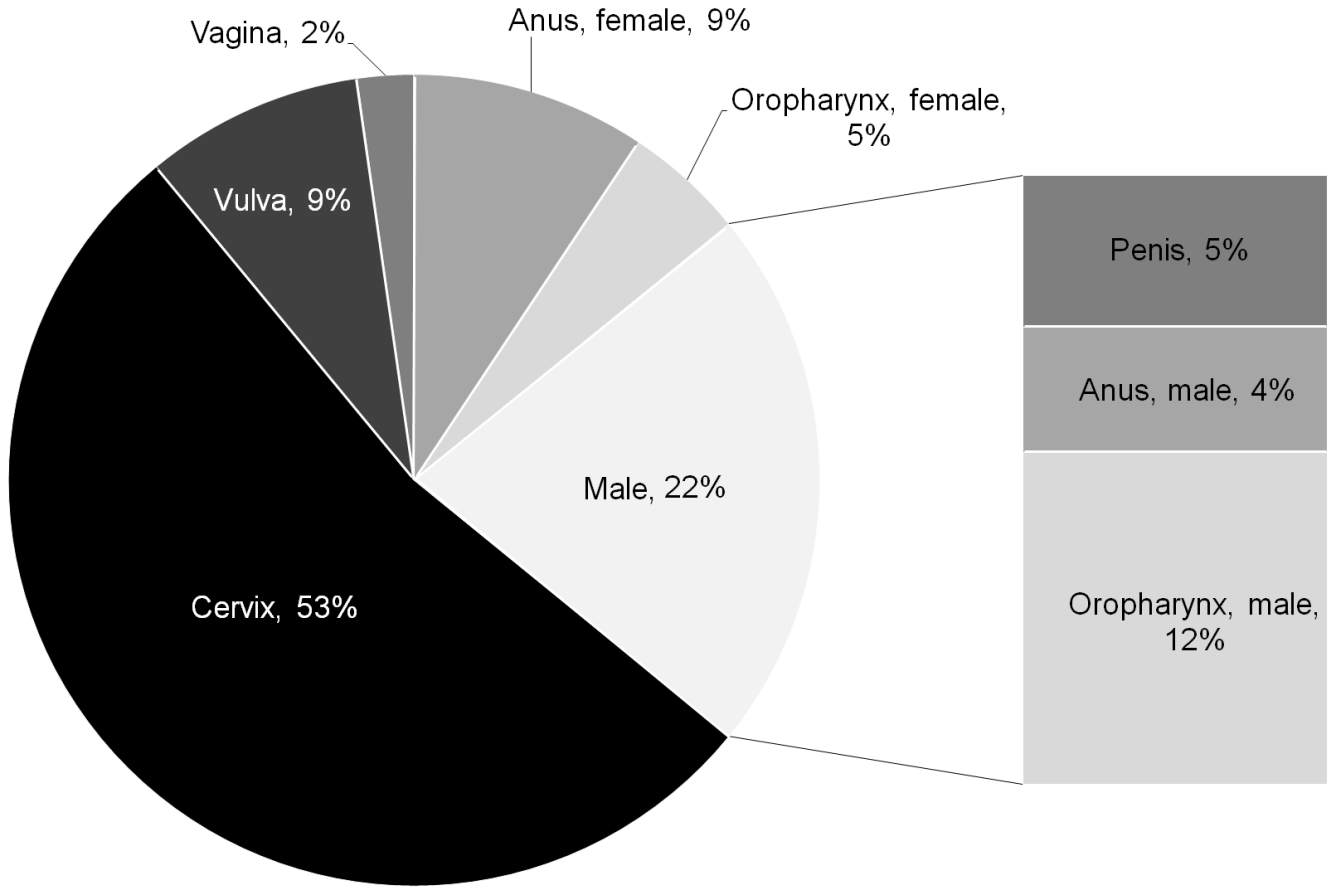
Proportion of human papillomavirus (HPV)-16 and -18 related cancers in Norway, by gender. For oropharyngeal cancers, we considered three sub-sites: 1) oropharynx, 2) base of tongue and 3) tonsils. For all other cancers, we considered all histologies reported at each sub-site. Percentages have been rounded to the nearest whole number.

Persistent infection with human papillomavirus (HPV), a known causal agent for cervical cancer, is emerging as an important risk factor for several diseases in both women and men. High-risk, oncogenic HPV infections, most importantly HPV-16 and to a lesser extent HPV-18, are responsible for a proportion of vulva, vaginal, anal, penile and oropharygeal cancers (**Figure 1**). Infection with low-risk HPV, most notably HPV-6 and -11, are responsible for the majority of genital warts and recurrent respiratory papillomatosis (RRP).

In Norway, a 3-dose schedule of the quadrivalent HPV vaccine, shown to have high efficacy against HPV-16, -18, -6, and -11, has been offered to pre-adolescent girls through school-based delivery in the 7th grade since 2009. The most recent cohort of girls (born in 1999) has achieved 2- and 3-dose coverage rates of 79% and 71%, respectively [1]. Worldwide, a growing number of countries recommend or permit HPV vaccination for males aged 9–26, though few have offered to publicly fund the policy [2]. Given the highly transmissible nature of HPV through sexual activity, high vaccination coverage among pre-adolescent girls may provide a high level of indirect benefit to boys, effectively reducing the burden of HPV-related disease in both sexes [3]. Ecological data from Australia and the U.S. support this finding [4], [5]. Generally, cost-effectiveness analyses evaluating HPV vaccine introduction in several countries have concluded that the incremental benefit of expanding HPV vaccination programs to include pre-adolescent boys may not justify the added cost, particularly if vaccination coverage among girls is high [6]–[8]. One of the most influential parameters in such analyses is vaccine price. In Norway, the market price is roughly $150 per dose; however, pharmaceutical statistics from Norway in 2011–2012 indicate that the nationally-negotiated tender may be half of the market price (i.e., approximately $75 per dose [9]). It is conceivable that future negotiations may continue to press the vaccine price down.

In light of declining vaccine prices and the growing evidence of vaccine impact on reducing multiple HPV-related conditions in both women and men [10], countries such as Norway must assess whether including boys in the publicly funded childhood vaccination program is warranted. In addition, alternate dosing schedules (i.e., two versus three doses) may further reduce the cost per vaccinated individual without decreasing efficacy [11]. A comprehensive analysis across a broad range of vaccine prices for developed countries has not been undertaken. In addition, evaluating the value of expanding the Norwegian HPV vaccination program to include boys has not been conducted but is essential for guiding setting-specific health care policy and is required inter alia for priority-setting in Norway [12]. We aim to assess whether HPV vaccination of pre-adolescent boys is a cost-effective use of finite resources by explicitly considering HPV transmission dynamics, including a wide range of HPV-related conditions, and exploring the impact of different vaccine tender prices.

## Methods

### Decision analytic approach

We adapted a dynamic model of HPV sexual transmission and multiple disease simulation models to reflect the health and economic burden of HPV-related conditions in Norway across multiple birth cohorts of men and women [7], [13]. We compared the current HPV vaccination program that targets only 12-year-old girls to an expanded program that includes 12-year-old boys. The analysis included outcomes related to the HPV types targeted by the quadrivalent vaccine, including carcinogenic types 16 and 18 and non-carcinogenic types 6 and 11. We simulated the female vaccination program starting in 2009, while male vaccination was assumed to be implemented in 2014; to isolate the impact of vaccinating boys, the costs and benefits for the first five years of the female-only program were not counted. Taking into consideration all HPV-related conditions, we projected long term outcomes across the entire lifetime of the first 30 male and female cohorts under a gender-neutral vaccination program compared to a female-only program. Monetary costs were measured in 2010 Norwegian Kroner (NOK) and converted to US dollars using the average annual 2010 exchange rate ($1 = NOK6.05) [14]. We adopted a societal perspective and discounted costs and health benefits by 4% per year over the lifetime of each simulated cohort, consistent with Norwegian guidelines [15]. We assessed cost-effectiveness by calculating the incremental cost-effectiveness ratio (ICER), defined as the additional cost divided by the additional quality-adjusted life year (QALY) gained associated with one strategy compared to the next less costly strategy. We used a commonly cited Norwegian threshold of NOK500,000 per QALY gained (≈$83,000) to represent a “cost-effective” intervention [16], but also considered alternative thresholds ($30,000–$100,000 per QALY) to reflect the lack of consensus for a single threshold value in Norway^15^ and a range of threshold values cited in other settings.

### Models

We refined a previously-developed dynamic model of HPV-16 and -18 transmission [7], [13] to simulate heterosexual behavior between men and women in Norway and an individual-based disease model [17] to simulate HPV-induced cervical cancer in the context of the current Norwegian screening program. For all non-cervical HPV-related conditions, we used an incidence-based modeling approach to capture the health and economic burdens in both genders.

The dynamic model is age-structured in yearly intervals and simulates multiple birth cohorts over their lifetimes. Individuals are designated into one of four sexual activity groups (i.e., none, low, moderate, high), which governs the rate of partner change per year and varies by age and gender, based on data from two Norwegian sexual behavior surveys [18], [19]. HPV transmission occurs as a function of the number of new partners, prevalence of HPV in the opposite gender, and HPV-type and gender-specific probabilities of transmission from an infected partner to an uninfected partner. We assumed that male-to-female transmission was 0.80 times as much as female-to-male transmission estimated from an empirical study [20]. After clearance from an initial HPV infection, partial gender- and type-specific immunity develops, reducing future rates of acquiring the same type of HPV.

The individual-based stochastic model, previously adapted to the Norwegian context [17], mimics the natural history of cervical cancer and allows for complex screening algorithms to be simulated. Individual girls enter the model and face age-specific monthly probabilities of acquiring HPV, categorized as 16, 18, other high-risk or low-risk types. Individuals can develop precancerous lesions, which may regress naturally, or progress to invasive cervical cancer. Survival from cervical cancer was estimated from the Cancer Registry of Norway and varied based on stage of detection [21].

Initial parameters for both models were based on data from epidemiological and demographic studies [7], [13], [22]. We calibrated the models using a likelihood-based method to fit empirical outcomes observed in Norway, such as HPV prevalence and cervical cancer incidence. Additional explanation of the Norwegian-specific calibration process can be found in (**File S1**). The natural history of HPV-related non-cervical conditions is not well known; therefore, we elected to develop simplified models simulating the disease incidence rates by age and gender [21] and attributable fraction of vaccine-targeted HPV types for each of these conditions [23], [24]. We used the transmission model to project the reductions in vaccine-type HPV incidence attributable to vaccination, including both direct and indirect protection (i.e., herd immunity). These reductions in HPV infections were used as inputs into the disease simulation models to then project the corresponding reductions in related diseases. For all models, individuals faced all-cause mortality at each time step, and when applicable, excess mortality after disease onset.

### Costs

Baseline costs associated with HPV vaccination included costs for all three vaccine doses using the estimated tender price of $75 per dose [9], wastage and supplies. We assumed that 10% [1] of those who initiate vaccine do not complete all three doses, thereby incurring some vaccine costs but no vaccine benefit; however, we examined alternative benefit assumptions in sensitivity analysis. Estimation of costs associated with cervical cancer screening, diagnosis, and treatment is documented in a previous cost-effectiveness analysis [17]. Norwegian-specific treatment costs associated with the other non-cervical HPV-related conditions included all direct medical and nonmedical costs associated with diagnosis, treatment and post-treatment surveillance, if applicable (**Table 1**). Future costs and benefits for juvenile-onset RRP were discounted to the time of vaccination of the mother. See (**File S1**) for further explanation of costing methods.

**Table 1:** Selected inputs.

HPV-related conditions (ICD-10 code)	Women	Men	Setting
Anal cancer (C21)			
Incidence per 100,000, mean (range)^a^	1.9 (0–9.1)	0.9 (0–5.7)	Norway [21]
5-year relative survival (%)^b^	70.4	51.3	Norway [21]
Quality of life adjustment^c^	0.57		Australia [26]
Cases attributable to HPV-16 (%)	73		N. Europe [23]
Cases attributable to HPV-18 (%)	9		N. Europe [23]
Cost per case ($)^d^	37,500		Norway^d^
Cervical cancer (C53)			
Incidence per 100,000, mean (range)^a^	24.0 (0–32.0)	–	Norway [21]
5-year relative survival (%)^b^	19.9–91.0	–	Norway [21]
Quality of life adjustment^c^	0.48–0.76	–	US [25]
Cases attributable to HPV-16 (%)	56	–	Norway [32]
Cases attributable to HPV-18 (%)	16	–	Norway [32]
Cost per case ($)^d^	25,800–59,600	–	Norway^d^
Oropharyngeal-related (C01,09,10)			
Incidence per 100,000, mean (range)^a^	1.5 (0–6.5)	3.8 (0–14.1)	Norway [21]
5-year relative survival (%)^b^	57.6	60.3	Norway [21]
Quality of life adjustment^c^	0.58		Australia [26]
Cases attributable to HPV-16, -18 (%)	53		Norway [24]
Cases attributable to HPV-16, -18 (%)	1		Norway [24]
Cost per case ($)^d^	49,000		Norway^d^
Penile cancer (C60)			
Incidence per 100,000, mean (range)^a^	–	2.0 (0–11.4)	Norway [21]
5-year relative survival (%)^b^	–	81	Norway [21]
Quality of life adjustment^c^	–	0.79	Australia [26]
Cases attributable to HPV-16 (%)	–	42	N. Europe [23]
Cases attributable to HPV-18 (%)	–	4	N. Europe [23]
Cost per case ($)^d^	–	17,500	Norway^d^
Vaginal cancer (C52)			
Incidence per 100,000, mean (range)^a^	0.6 (0–4.3)	–	Norway [21]
5-year relative survival (%)^b^	48.6	–	Norway [21]
Quality of life adjustment^c^	0.59	–	Australia [26]
Cases attributable to HPV-16 (%)	63	–	N. Europe [23]
Cases attributable to HPV-18 (%)	3	–	N. Europe [23]
Cost per case ($)^d^	26,400	–	Norway^d^
Vulvar cancer (C51)			
Incidence per 100,000, mean (range)^a^	3.4 (0–26.5)	–	Norway [21]
5-year relative survival (%)^b^	72.8	–	Norway [21]
Quality of life adjustment^c^	0.65	–	Australia [26]
Cases attributable to HPV-16 (%)	38	–	N. Europe [23]
Cases attributable to HPV-18 (%)	6	–	N. Europe [23]
Cost per case ($)^d^	27,900	–	Norway^d^
**Non-cancer HPV related conditions**			
Genital warts			
Incidence per 1,000, (age-specific range)	0.02–7.14	0.01–8.85	Sweden [33], UK [34]
Quality of life adjustment^c^	0.9277		UK [31]
Cases attributable to HPV-6, -11 (%)	90		Multiple [35],
Cost per case ($)^d^	400		Norway^d^
Juvenile recurrent respiratory papillomatosis			
Incidence per 100,000	0.17		Norway [37]
Quality of life adjustment^c^	0.69		US [30]
Cases attributable to HPV-6, -11 (%)	100		Multiple [35]
Cost per case ($)^d^	133,800		Norway^d^

HPV: human papillomavirus,

a Mean incidence reported for 2008–2010 for all HPV-related cancers except cervical cancer. Variation represents range in age-specific rates. Invasive cervical cancer incidence (used for calibration) is reported based on the pre-screening (1953–1969) mean of the minimum and maximum annual incidence from Norwegian Cancer Registry.

b 5-year relative survival is reported for calendar-period observation for 2006–2010; for cervical, the range represents stage-specific estimates for local (91%), regional (66%), and distant (19.9%).

c Quality of life adjustment range from a health state utility weight of 0 (death) to 1 (perfect health). Weights for cervical cancer varied according to stage (local: 0.76 for five years; regional: 0.67 for five years; distant: 0.48 five years). Utility weights for other non-cervical HPV-related cancers are applied for five years. For genital warts, a mean quality of life loss of 6.6. days is assumed [32], which is approximately a utility weight of 0.9277 over three months; for recurrent respiratory papillomatosis, health state utility weight of 0.68 over four years is assumed. Disease specific utility weights were multiplied to baseline age-specific utility weights [29] to estimate overall utility.

d Cost per case is expressed in 2010 US dollars (1 USD = 6.05 Norwegian Kroner) and represent discounted (4% per year) costs for diagnosis and 5-year follow-up inclusive of direct (procedures, inpatient stays, general practitioner visits) and non-direct medical costs (transport) and patient time. The proportion of direct non-medical costs for all non-cervical conditions was estimated from cervical cancer (15%) and applied to baseline direct medical costs. Treatment of cervical cancer varies according to stage of detection (local: $25,800; regional: $51,600; distant: $59,600). See (**File S1**) for estimation methods.

### Health-related quality of life

Health state utility weights for cervical cancer varied according to stage (**Table 1**) [25]. For non-cervical cancers, we opted to use a study that elicited utility values for multiple non-cervical cancers simultaneously. Valuations were elicited using standard gamble from the general population in Australia [26]. In Norway, the long-term impact after surviving a gynecological cancer (average of 12 years) on quality of life has been shown not to differ from the general public [27]. Furthermore, a Danish study that followed women with advanced stage cervical cancer found that quality of life among women 18-months post radiation treatment was comparable to the general population [28]. Based on these data, we conservatively assumed that individuals with detected cancer remained in a state of reduced quality of life for five years, after which individuals returned to their gender- and age-specific utility values elicited from the general population in a neighboring Scandinavian country [29]. For HPV-6 and -11 related conditions, we applied disease-specific utility values for the average duration of the disease (i.e., 3-months for genital warts and 4.2 years for RRP) [30], [31].

### Other model inputs

We synthesized available data from Norway, or from surrounding countries when Norwegian-specific data were not available, to inform parameter inputs, such as disease incidence, survival and cases attributable to vaccine-targeted HPV types (**Table 1**). Our base case assumed vaccine efficacy against disease outcomes related to vaccine-targeted HPV types of 100% for females and 90% for males over the lifetime, in line with a recent systematic review [10]. Additional information may be obtained from the authors upon request.

### Analysis

We compared a scenario of routine HPV vaccination of 12-year-old girls only at the current Norwegian 3-dose coverage level (71%) to a scenario that assumes similar coverage is achieved by 12-year-old boys. We calculated the health outcomes and costs of the different HPV-related conditions and explored the impact of different vaccine prices ranging from $20–$160 per dose. We evaluated the impact of model assumptions on cost-effectiveness using one- and multi-way sensitivity analysis. For one-way sensitivity analysis, we varied vaccine efficacy, duration, incidence of oropharyngeal cancer and considered an alternate vaccine dose schedule (assuming two doses confer the same vaccine protection as three doses). To provide an approximate estimate of the impact of the men who have sex with men (MSM) population on results, we systematically reduced the herd immunity benefits conferred to the male-population in the female-only vaccination strategy. For the multi-way sensitivity analysis, we simultaneously varied treatment costs and the attributable fraction of HPV-16 and -18 in each HPV-related condition to determine “optimistic” and “pessimistic” results. We also varied analytic assumptions, such as the discount rate (0% and 3%) and consideration of direct costs only, consistent with Norwegian guidelines [15]. Lastly, we considered a third scenario which involved increasing the coverage rate among pre-adolescent girls to 90%, the level currently achieved by the measles, mumps, rubella (MMR) vaccine administered to pre-adolescent Norwegians aged 11–12. Expanding HPV vaccination coverage for girls was directly compared to extending coverage to boys in order to determine which strategy minimizes the burden of HPV-related conditions in Norway at a reasonable cost.

## Results

### Epidemiological outcomes

Assuming the current 3-dose vaccination coverage rate among pre-adolescent girls remains constant at 71% with 100% lifelong efficacy, the girls-only vaccination program was projected to substantially reduce future cancer incidence (**Table 2**). The additional reductions in cancer incidence by adding male vaccination (assuming equal coverage) were modest. We project that, for the same future cohort, female genital warts may decrease by 77% and male genital warts may decrease by 62%, under a female-only vaccination program. For a gender-neutral vaccination program, reductions in genital warts may increase to 85% and 84% among females and males, respectively.

**Table 2:** Projected reductions in HPV-related cancer incidence, by gender.

	No vaccination	Girls-only vaccination^b^	Girls + boys vaccination^b^
Disease, 2008–2010^a^	Incidence rate	Change in incidence rate compared to no vaccination (% reduction)	Change in incidence rate compared to girls vaccination (% reduction)
Female			
Cervical^c^	12.6	−5.2 (41%)	−0.8 (10%)
Vulvar	3.4	−1.2 (36%)	−0.1 (6%)
Vaginal	0.6	−0.3 (54%)	−0.03 (11%)
Anal	1.9	−1.3 (67%)	−0.1 (21%)
Oropharyngeal	1.5	−0.6 (43%)	−0.1 (9%)
Male			
Penile	2.0	−0.6 (29%)	−0.3 (18%)
Anal	0.9	−0.5 (52%)	−0.2 (46%)
Oropharyngeal	3.8	−1.0 (33%)	−0.6 (22%)

a Age-standardised incidence rates are expressed as 100,000 per individual and have not been adjusted for world population; rates under no vaccination scenario refer to current rates reported from the Cancer Registry of Norway [21]

b Projections reflect the expected cancer reduction estimated from the dynamic transmission model for the last cohort to be vaccinated in this analysis. See Methods section for assumptions regarding vaccine efficacy against non-cervical cancers.

c Projected reduction in risk of cervical cancer is estimated from the stochastic disease model and in the context of current cervical cancer screening compliance.

### Cost-effectiveness

**Figure 2:**
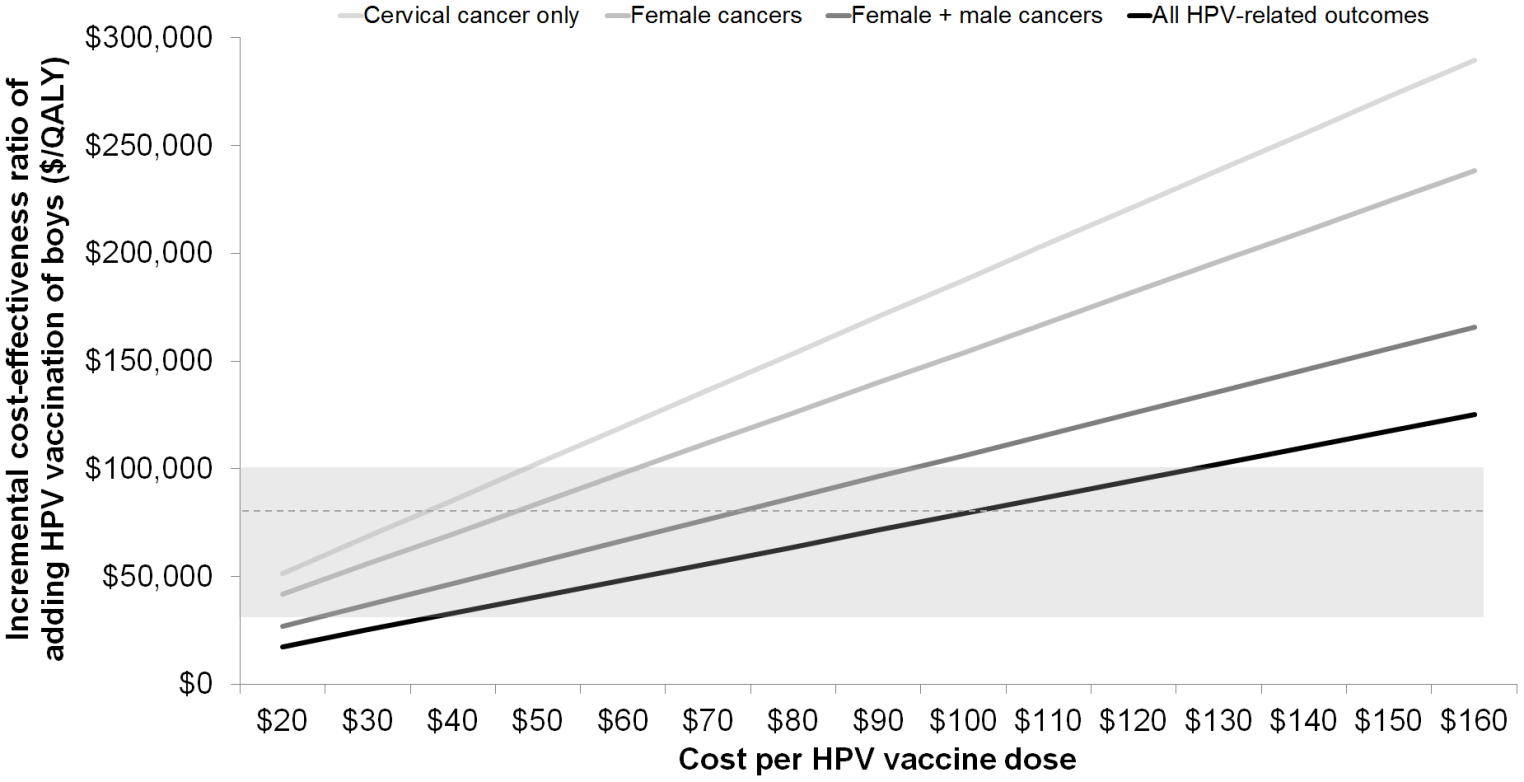
Incremental cost effectiveness ratios (ICER) of vaccinating pre-adolescent girls and boys compared to vaccinating pre-adolescent girls only. Shaded area represents the broad range of willingness-to-pay thresholds ($30,000–$100,000 per QALY gained) accepted across developed countries. Dotted line represents a threshold often cited in Norway ($83,000 per QALY gained).^16^ Cost per dose excludes the administration cost (≈$14 per dose).

At the assumed tender price of $75 per dose, the cost per QALY gained from routine vaccination of girls only (compared to no vaccination) was $20,600 when including only benefits related to cervical outcomes and $5,000 when including benefits associated with all female and male HPV-related conditions (**Table 3**). Expanding the vaccination program to include pre-adolescent boys, assuming the same 3-dose coverage rate and 90% lifelong vaccine efficacy in males, the cost per QALY gained was $145,500 accounting for cervical cancer outcomes only, but fell to $60,100 per QALY gained when including all HPV-related outcomes. The incremental cost-effectiveness ratios of vaccinating both genders compared to vaccinating girls only over a wide range of vaccine prices is shown in **Figure 2**. Accounting for all HPV-related outcomes, expanding HPV vaccination to boys would be considered cost-effective at a vaccine cost per dose of approximately $101, $62 and $36 for willingness-to-pay thresholds of $83,000, $50,000 and $30,000 per QALY gained, respectively. Therefore, at the current market price, expanding the current HPV vaccination program to include boys would not be considered ‘good value for money.’ When restricting vaccine benefit to only cancers (i.e., no genital warts or RRP), the vaccine cost per dose would have to be at least 30% lower for male vaccination to be considered cost-effective, compared to girls-only vaccination.

**Table 3:** Incremental cost-effectiveness ratios of including pre-adolescent boys in the childhood vaccination program compared to vaccination of pre-adolescent girls only.

	Vaccination strategy^a^	
HPV-related outcome(s) included	Girls only^b^	Girls + boys^c^
Cervix only	$20,600	$145,500
Female cancers^d^	$12,800	$119,300
Female + male cancers^e^	$8,900	$81,700
All HPV-related conditions^f^	$5,000	$60,100

a Assumes a cost per dose of $75, exclusive of the administration cost (≈$14 per dose).

b Compared to no vaccination.

c Compared to girls-only vaccination.

d Includes female cervical, vulvar, vaginal, anal and oropharyngeal cancers,

e Includes male anal, oropharyngeal and penile cancers,

f Includes cervical, vulvar, vaginal, anal, oropharyngeal and penile cancers related to HPV-16, -18, and genital warts and recurrent respiratory papillomatosis related to HPV-6, -11.

### Sensitivity analysis

The impact of model assumptions on the incremental cost-effectiveness ratio of a gender-neutral vaccine program compared to a girls-only program (including outcomes related to all HPV-related conditions) for three vaccine prices is shown in **Table 4**. The incremental cost-effectiveness ratios associated with including boys in the vaccination program (at $75 per dose) begin to exceed a threshold of $83,000 per QALY either when the cost-effectiveness results were expressed in terms of life years (not QALY) gained or when the lower bound of the disease-specific HPV-16 and -18 attributable fractions and lower disease treatment costs (“pessimistic scenario”) were assumed simultaneously. At the market price of the vaccine ($150 per dose), vaccination of both genders was never cost-effective across key parameter variations given a threshold of $83,000 per QALY gained. The incremental cost-effectiveness ratio only fell below $100,000 per QALY gained when we considered the benefit and costs associated with a 2-dose vaccination schedule or lower discount rates. At $50 per dose, the ratios generally remained above $30,000 per QALY gained. For a vaccine price of $75 per dose, we found that the cost per QALY gained fell below $50,000 only when the herd immunity benefits conferred to the male-population in the girls-only vaccination program was reduced by more than 15% (i.e., assuming the female-only HPV vaccination program produced smaller herd immunity benefits due to the MSM population). When we doubled the incidence of oropharyngeal cancer in both genders, we found that the incremental cost-effectiveness ratios of vaccinating boys fell by approximately 15–17%, depending on the cost per dose of the vaccine.

**Table 4:** Impact of parameter assumptions on the cost-effectiveness of including boys in a vaccination program against human papillomavirus (HPV) (including all HPV-16,-18,-6,-11 related conditions).

	Cost per dose^a^		
	$50	$75	$150
**Girls only vaccination (cost per QALY gained)** b			
Base case	$1,600	$5,000	$14,600
Vaccine duration: 20 yrs	$6,500	$12,000	$27,700
Direct medical costs only	$2,680	$6,030	$15,650
No disease-specific utilities	$5,500	$10,000	$23,000
Discount rate 0%	Cost saving	Cost saving	Cost saving
Discount rate 3%	Cost saving	$1,600	$7,550
2-dose schedule (79% coverage)^c^	Cost saving	$600	$7,000
Double oropharygeal cancer	$800	$3,800	$12,200
Optimistic scenario analysis^d^	Cost saving	$2,100	$10,100
Pessimistic scenario analysis^e^	$3,100	$6,600	$16,800
**Girls + boys vaccination (cost per QALY gained)** f			
Base case	$40,400	$60,100	$116,700
60% boys coverage	$44,400	$65,800	$127,200
80% boys vaccine efficacy	$56,100	$82,300	$157,400
Vaccine duration: 20 yrs	$38,300	$57,200	$111,400
Direct medical costs only	$41,630	$61,370	$118,500
No disease-specific utilities	$67,900	$98,500	$186,500
Discount rate 0%	$1,490	$4,080	$11,500
Discount rate 3%	$23,680	$36,240	$72,300
Increasing girls coverage: 90%^g^	Dominated	Dominated	Dominated
2-dose schedule (79% coverage)^c^	$27,680	$42,320	$84,330
Double oropharygeal cancer	$33,300	$50,200	$98,700
Optimistic scenario analysis^d^	$37,100	$56,300	$111,600
Pessimistic scenario analysis^e^	$63,100	$91,700	$174,000

QALY: Quality-adjusted life year.

a All costs are expressed in 2010 US dollars (1US$ = NOK6.05) and rounded to the nearest $10,

b Compared to no vaccination,

c The 2012 2-dose coverage for girls in Norway is 79%, this scenario assumes boys achieve the same 2-dose coverage and vaccine efficacy is equal to 3-doses.

d Optimistic scenario analysis: Upper bound of HPV-16, -18 attributable fraction and upper bound of treatment cost,

e Pessimistic scenario analysis: Lower bound of HPV-16, -18 attributable fraction and lower bound of treatment costs,

f Compared to girls-only vaccination.

g Assumes HPV vaccination requires 3 doses and girls achieve a similar coverage as the MMR vaccine (administered age 12–13 years in Norway). Increasing coverage among girls to 90% was more beneficial and less costly than (i.e., dominated) adding boys with 71% coverage.

**Figure 3:**
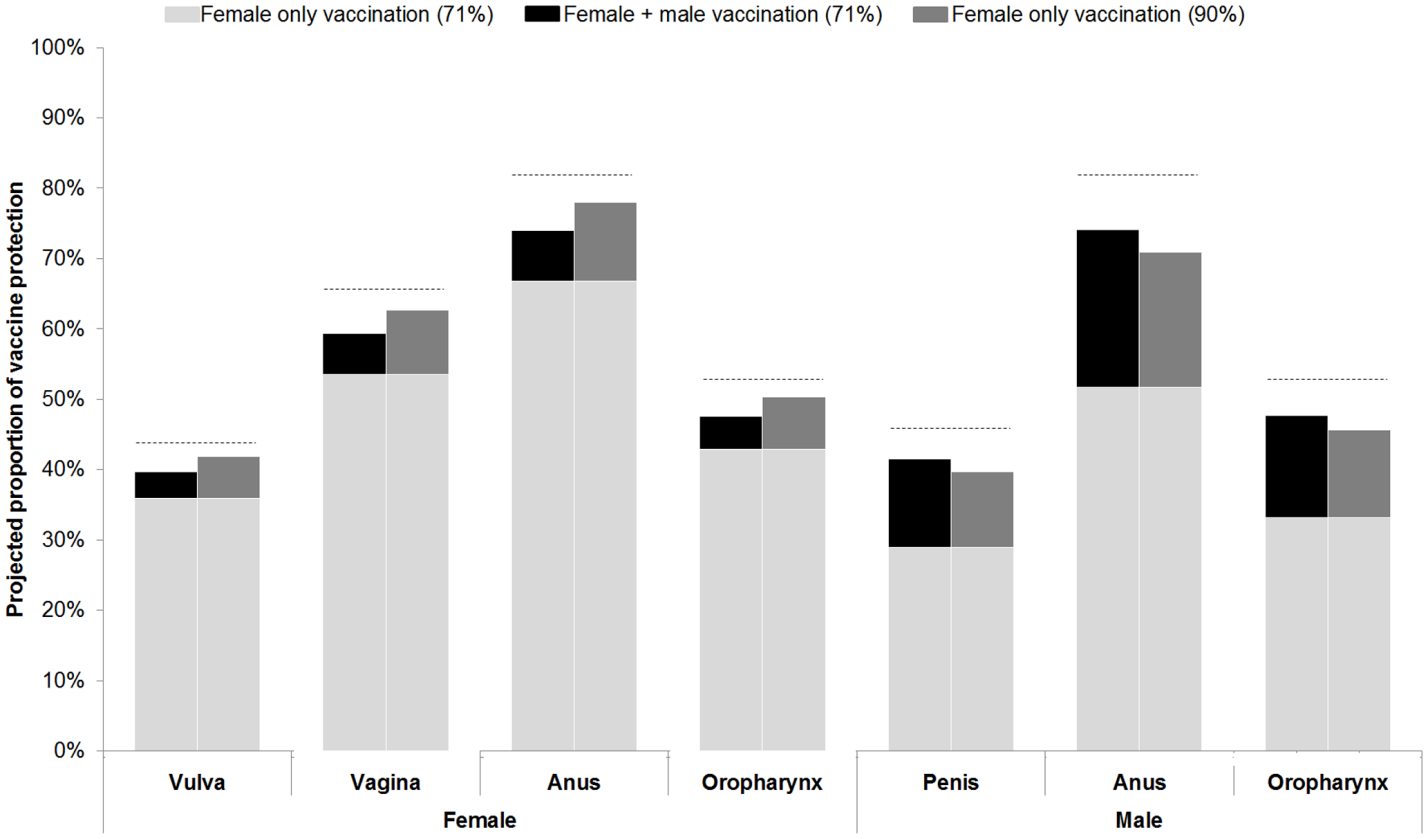
Projected impact of vaccinating both pre-adolescent girls and boys at 71% coverage compared to increasing coverage to 90% for a girls-only program on non-cervical human papillomavirus (HPV)-16, -18 related cancers. Dotted lines represent the theoretical maximum attributable fraction of HPV-16, -18 for each condition.

Apart from vaccine price, the discount rate and increasing vaccination coverage in pre-adolescent girls had the most influence on results. For example, increasing vaccination coverage of girls alone to 90% was more effective and less costly, and therefore dominated, a scenario of vaccinating both genders with 71% coverage. We calculated that more than twice the amount per vaccinated girl – or six times the amount, if the funds were targeted specifically to those who did not previously uptake – could be spent before adding boys to the vaccination program would be equally cost-effective as increasing participation among girls only. Although extending HPV vaccination to boys provides benefits to both genders, increasing coverage within a girls-only program prevents more HPV-16,-18 related female cancers than a gender-neutral program that achieves 71% coverage. Through additional herd immunity benefits, increasing female-only vaccine uptake can prevent nearly as many HPV-related cancers among men as by vaccinating boys directly (**Figure 3**). Even if increasing coverage among girls did not provide any additional herd immunity benefits to the boys, the scenario still provided greater overall reductions in cancer cases than vaccinating both genders (see **Table S9 in File S1**).

## Discussion

Our findings confirm that vaccine price is one of the most influential parameters when determining cost-effectiveness of extending the current female-only HPV vaccination program to include boys. In order to aid policy decisions in settings where stakeholders are privy to the national tenders procured at a lower price per dose than the publicly available price, we express the incremental cost-effectiveness ratios across a range of plausible vaccine prices. Our analysis suggests that there may be some combinations of vaccine price and willingness-to-pay thresholds where adding boys is cost-effective, even when current 3-dose coverage rates are already high (i.e., 70% among girls).

In our base case scenario, which considers a realistic Norwegian vaccine tender price of $75 per dose, we found that adding 12-year-old boys to the current HPV vaccination program may be considered ‘good value for money’ at a willingness-to-pay threshold of $83,000 per QALY gained. However, the most recent Norwegian guidelines for economic evaluation emphasize that consensus surrounding a single Norwegian threshold value has not been established [15]. In addition, there is support for a change in screening guidelines for unvaccinated women to a 6-year interval with primary HPV testing (for women aged 34 years or older) [38], a strategy estimated at approximately $30,000 for each additional year of life saved [17]. At this lower willingness-to-pay threshold, it would not be considered cost-effective to expand the current program, unless the vaccine price was less than $36 per dose (**Figure 2**). At a price of $120 to $150 per dose, expanding the HPV vaccination program to include boys is unlikely to be cost-effective even when considering the higher threshold value ($83,000 per QALY gained), a finding that is generally consistent with other studies [6]–[8]. Of note, the Norwegian Ministry of Health has approved cancer medications for reimbursement at threshold values beyond $83,000 per QALY gained; however, the total budget impact of these pharmaceuticals is often small as the targeted health conditions are relatively uncommon [39]. The same cannot be said for expanding a childhood vaccination policy to include all boys, which would essentially double the current HPV vaccination budget.

To our knowledge, there are only three other studies that have assessed the potential value of adding boys to the pre-adolescent HPV vaccination program that simultaneously account for HPV transmission dynamics, consider all HPV-related outcomes, and report results in terms of cost-effectiveness [6], [7], [40]. Other studies, however, have addressed epidemiological endpoints and the incremental benefit of adding boys to the vaccination program using static or dynamic models considering one or more HPV-related outcomes [3], [8], [29], [41]–[47]. The importance of certain assumptions for model structure (particularly for transmission dynamics and non-cervical HPV-related conditions), natural immunity, coverage and costs have been discussed previously [48]. A recent U.S.-based study concluded that for lower coverage rates (20–30%) among girls, adding vaccination of boys becomes an attractive policy, but if baseline coverage is 75% among girls, the incremental cost-effectiveness ratio exceeds $100,000 per QALY gained [6]. This study, however, did not consider the impact of vaccine price per dose of less than $120. Male HPV vaccination in Norway may be more attractive than those found in other settings due to several reason that include (but are not limited to) the comparatively higher prevalence of HPV-16 and -18 infections reported in Norway, higher attributable fraction of HPV-16 and -18 in oropharyngeal cancers [24], the higher baseline burden of disease (pre-vaccination), and higher Norwegian labor costs that may contribute to higher direct medical and non-medical treatment costs. In addition, we used health-related quality of life estimates reported by Conway and colleagues [26] and with the exception of penile cancer, these estimates are consistently lower than those reported and used in other studies.

For a specified vaccine price, our findings were generally stable to variations in critical parameters, with the notable exception of considering a scenario in which we compared expanding vaccination to boys versus increasing the coverage rate among girls, consistent with another study [6]. If feasible, higher uptake in girls may lead to further reduction in the total burden of HPV-related diseases, even considering an extreme scenario where increasing girls' coverage did not yield any further herd immunity benefits in males. Another modeling study showed that the most effective strategy to reduce population prevalence is by optimizing coverage in a single-sex vaccination program [47]. In addition, the feasibility of achieving 71% coverage among males, in whom the disease burden is considerably less than in females (**Figure 1**), also requires consideration. On the other hand, overall vaccine acceptability with a gender-neutral policy may increase without additional investments, resulting in higher coverage among girls. When we considered an alternative dosing schedule (using optimistic assumptions surrounding vaccine duration and efficacy), we found that a 2-dose regimen resulted in one of the most appealing strategies for vaccinating boys; however, there is substantial uncertainty with respect to the duration of protection from two doses [11]. As expected for programs with large upfront costs, the discount rate for vaccination programs that avert future disease was particularly impactful and should be taken into consideration when interpreting the results of a long-term cost-effectiveness analysis of preventative programs.

Finally, as both genders are responsible for HPV transmission, one may argue on equity grounds that both genders should get vaccinated to share the burden in reducing the risk of HPV-related disease, as well as have equal access to direct vaccine benefits. Equity versus efficiency arguments should be considered along-side the decision-making process and are particularly relevant in Norway where guidelines explicitly emphasize this trade-off [15].

### Limitations

Limitations of our modeling approach have been previously discussed [7], [13], but some deserve particular consideration. Simplifying assumptions were inherently necessary due to data limitations or modeling constraints. For example, we assumed that the burden of HPV-related diseases remain constant over time while evidence suggests the incidence of oropharyngeal cancer related to HPV may be increasing [49]. When we considered this possibility, we found vaccinating boys to be more attractive, but the overall conclusions of the analysis were stable. Alternatively, we also did not account for the better prognosis among HPV-positive cancers compared to their HPV-negative counterparts, potentially overestimating vaccine benefit. We modeled heterosexual behavior while transmission among MSM was not explicitly considered. Although the burden of disease estimates did reflect cases among all individuals (including MSM), this omission likely overestimated the level of herd immunity conferred to males in a female-only vaccination program. Even so, we found that the herd immunity benefits in the female-only HPV vaccination program would have to be overestimated by more than 15% in order for the cost per QALY gained to fall below $50,000. Norwegian sexual behavior data suggest that the proportion of MSM is between 0.6% and 2.8% (depending on age) whereby more individuals identify with bisexual behavior compared to exclusively being homosexual, particularly prior to age 30, when the majority of HPV transmission takes place (see **File S1**). While a small proportion of herd immunity may be overestimated in our model, bisexual behavior may continue to propagate herd immunity benefits and the expected herd immunity reduction with at-most a 3% exclusively-male MSM population would be less than the threshold of 15%.

We did not account for any level of vaccine cross-protection related to non-vaccine types observed in clinical trials [50]. The duration of cross-protection is uncertain, and the majority of male HPV-related diseases are attributed to HPV-16 and -18, so the inclusion of cross-protection is likely to be nominal in reducing the burden of disease among males. Inclusion of cross-protection may reinforce the argument for increasing female coverage rate, however.

The quality and completeness of the Norwegian Cancer Registry have been documented [51], but little research has been done on the burden of HPV infection in Norway, particularly in men, or more recent sexual mixing patterns by age and by sexual activity. We used empirical data from one large city in Norway to inform our bounds for HPV prevalence in Norway (Mari Nygård, personal communication), but there may be considerable geographic variation with respect to sexual behavior and HPV prevalence. In order to fit the observed data, our calibrated transmission probabilities may have been higher in order to fit a high observed prevalence. Conversely, we did not allow for the potential of cross-border behavior, which may overestimate herd immunity, given vaccination rates among girls may not be as high in other countries. Lastly, our understanding of the natural history and HPV type attribution of non-cervical HPV-related diseases is limited but growing; analyses can be revisited as new detection methods and systematic reviews continue to define the natural history and attributable fraction of HPV on associated cancers.

## Conclusions

At Norway's assumed HPV vaccine tender price, vaccinating boys seems attractive and may warrant a change in the current female-only vaccination policy. However, increasing coverage in girls is uniformly more effective and cost-effective than expanding vaccination coverage to boys and should be considered a priority.

## Supporting Information

File S1 Supplementary appendix providing additional information on model inputs, the Norwegian-specific calibration process, and additional results.
(DOCX)
