# Supplementary material for: Prevention of HPV-Related Cancers in Norway: Cost-Effectiveness of Expanding the HPV Vaccination Program to Include Pre-Adolescent Boys
Source: PLoS One. 2014 Mar 20;9(3):e89974. doi: 10.1371/journal.pone.0089974 (PMC3961226; doi:10.1371/journal.pone.0089974)
Supplement: File S1 — Supplementary appendix providing additional information on model inputs, the Norwegian-specific calibration process, and additional results. (DOCX) [file pone.0089974.s001.docx]

**To accompany manuscript:**

Prevention of HPV-related cancers in Norway: Cost-effectiveness of expanding the HPV vaccination programme to include pre-adolescent boys

Authors:

Emily A Burger

Stephen Sy

Mari Nygård

Ivar Sonbo Kristiansen

Jane J Kim

**PART I: MODEL INPUTS AND CALIBRATION**

**PART II: COSTS**

**Part III: ADDITIONAL RESULTS**

**PART I:** MODEL INPUTS AND CALIBRATION

**Models**

We adapted a dynamic model of human papillomavirus (HPV)-16 and -18 transmission^1;2^ to simulate sexual behavior between heterosexual males and females in Norway. We incorporated new information on the transmission dynamics and natural history of HPV in men. For example, we assumed that compared to women, men do not develop the same level of natural immunity following the first HPV infection and clearance.^3^ In addition, the probability of a female-to-male transmission is believed to be greater than the converse due to higher viral load in the cervix compared to the male genitals.^4^ Assumptions and inputs to the dynamic model are discussed in greater detail below.

We used a stochastic disease model, to simulate HPV-induced cervical cancer in the context of the current Norwegian screening program. Initial parameters for both models were based on data from epidemiological and demographic studies and calibrated using a likelihood-based method to fit empirical outcomes, such as HPV prevalence, HPV type distribution and pre-screening cervical cancer incidence, observed in Norway. For all other non-cervical HPV-related conditions, we used an incidence-based modeling approach to capture the health and economic burdens for both genders.

**Epidemiological inputs**

Inputs such as age-specific fertility rates and population statistics were elicited from Statistics Norway (Table S1).^5^

Table S1. Population (and fertility rates), by sex and age, 2011

|  | **Females** | **Males** | **Fertility rate,**  **Annual per woman (2011)** |
| --- | --- | --- | --- |
| **0-4 years** | 151,104 | 159,663 | -- |
| **5-9 years** | 147,344 | 153,725 | -- |
| **10-14 years** | 152,033 | 159,706 | -- |
| **15-19 years** | 157,162 | 167,828 | 0.0071 |
| **20-24 years** | 162,243 | 168,352 | 0.0543 |
| **25-29 years** | 157,834 | 164,358 | 0.1207 |
| **30-34 years** | 159,003 | 166,836 | 0.1239 |
| **35-39 years** | 170,840 | 180,495 | 0.0578 |
| **40-44 years** | 181,858 | 191,792 | 0.0109 |
| **45-49 years** | 170,066 | 181,291 | 0.0006 |
| **50-54 years** | 157,553 | 165,360 | -- |
| **55-59 years** | 150,838 | 154,425 | -- |
| **60-64 years** | 141,462 | 144,685 | -- |
| **65-69 years** | 125,538 | 123,568 | -- |
| **70-74 years** | 88,034 | 79,090 | -- |
| **75-79 years** | 72,174 | 58,027 | -- |
| **80-84 years** | 64,041 | 43,877 | -- |
| **85-89 years** | 48,159 | 25,565 | -- |
| **90-94 years** | 23,604 | 8,781 | -- |
| **95-99 years** | 5,492 | 1,328 | -- |

**Sexual behavior**

We defined the level of sexual activity (number of partners in the previous 12 months) by four sexual activity groups (SAGs): None (0 partners), Low (1-2 partners), Moderate (3-4 partners) and High (5 or more partners). A study on sexual behavior among Norwegian adolescents aged 13-19^6^ and individual level data from the 2002 Norwegian population survey for ages 18-49^7^ were used to derive age-specific proportions in each SAG (Table S2).

Table S2. Proportion of females and males in each sexual activity group, by age

| **Age** | **None** | **Low 1-2** | **Moderate 3-4** | **High 5+** |
| --- | --- | --- | --- | --- |
| Females |  |  |  |  |
| 13-14 yrs | 0.8400 | 0.0800 | 0.0800 | 0.0000 |
| 15-17 yrs | 0.5633 | 0.3267 | 0.0800 | 0.0300 |
| 18-19 yrs | 0.3056 | 0.4861 | 0.1250 | 0.0833 |
| 20-24 yrs | 0.1149 | 0.7368 | 0.0861 | 0.0622 |
| 25-29 yrs | 0.0558 | 0.8290 | 0.0855 | 0.0297 |
| 30-34 yrs | 0.0609 | 0.8631 | 0.0532 | 0.0228 |
| 35-39 yrs | 0.0571 | 0.8810 | 0.0476 | 0.0143 |
| 40-49 yrs | 0.0825 | 0.8686 | 0.0412 | 0.0077 |
| Males |  |  |  |  |
| 13-14 yrs | 0.7600 | 0.1600 | 0.0800 | 0.0000 |
| 15-17 yrs | 0.6700 | 0.2200 | 0.0800 | 0.0300 |
| 18-19 yrs | 0.3684 | 0.4386 | 0.0877 | 0.1053 |
| 20-24 yrs | 0.1843 | 0.5877 | 0.1754 | 0.0526 |
| 25-29 yrs | 0.0711 | 0.7396 | 0.1420 | 0.0473 |
| 30-34 yrs | 0.0808 | 0.7828 | 0.0657 | 0.0707 |
| 35-39 yrs | 0.0710 | 0.8258 | 0.0516 | 0.0516 |
| 40-49 yrs | 0.0995 | 0.8094 | 0.0552 | 0.0359 |

For the youngest age groups, only data on the proportion of sexual debut were available (could not define SAGs or number of partnerships); therefore, we elected to distribute the proportion of sexually active13-14 year old females evenly among the low and moderate SAGs. For males, we held the proportion in the moderate SAG equal to females and distributed the remaining in the low SAG. We tested this assumption during model calibration and it was found to achieve the best model fit. We assumed one and two new partnerships per year for the female SAGs low and moderate, respectively. Gender, SAG and age-specific rates of new partnership acquisition were set as reported by the women; male rates were then adjusted to equalize and balance partnerships (essentially adjusting reported male new partnerships by 0.94). After adjustments and assumptions, the average number of lifetime partners came to approximately 8.06 and the empirical data from Norway (ages 18-49)^7^ estimates the number at 7.68 (standard deviation: 2.34) and 10.59 (standard deviation: 14) for females and males, respectively (Table S3). We assumed moderately assortative mixing by both age and by SAG using a mixing coefficient of 0.5 on a 0 to 1 scale (0 is equivalent to partnerships only formed with individuals from the same age or SAG; 1 is equivalent to partnerships formed at random). This assumption was also tested during calibration and found to achieve the best fit to Norwegian empirical data for age-specific HPV16 and HPV18 prevalence.

Table S3. Mean number of new partnerships from the opposite sex, in the last 12 months

| **Age** | **None** | **Low, 1-2** | **Moderate, 3-4** | **High, 5+** |
| --- | --- | --- | --- | --- |
| Females (number of male partners) | | | | |
| 10-14 yrs | 0.00 | 1.00 | 2.00 | 0.00 |
| 15-19 yrs | 0.00 | 1.08 | 2.53 | 6.00 |
| 20-24 yrs | 0.00 | 2.12 | 4.34 | 7.31 |
| 25-29 yrs | 0.00 | 0.81 | 3.26 | 10.00 |
| 30-34 yrs | 0.00 | 0.56 | 3.07 | 8.80 |
| 35-39 yrs | 0.00 | 0.46 | 3.30 | 5.00 |
| 40-49 yrs | 0.00 | 0.41 | 3.06 | 7.33 |
| 45-49 yrs | 0.00 | 0.41 | 3.06 | 7.33 |
| Males (number of female partners) | | | | |
| 10-14 yrs | 0.00 | 0.94 | 1.89 | 0.00 |
| 15-19 yrs | 0.00 | 1.10 | 2.42 | 5.54 |
| 20-24 yrs | 0.00 | 1.06 | 3.21 | 5.82 |
| 25-29 yrs | 0.00 | 0.89 | 3.15 | 6.26 |
| 30-34 yrs | 0.00 | 0.56 | 2.62 | 8.50 |
| 35-39 yrs | 0.00 | 0.46 | 3.19 | 6.61 |
| 40-44 yrs | 0.00 | 0.35 | 3.12 | 6.83 |
| 45-49 yrs | 0.00 | 0.35 | 3.12 | 6.83 |

The model does not explicitly account for men who have sex with men (MSM), and may overestimate herd immunity benefits. However, some herd immunity benefits will still be experienced among those with bisexual tendencies. Norwegian sexual behaviour data suggest that the proportion of MSM ranges from 0.6-2.8% (depending on age) while more individuals identify with bisexual behaviour compared to exclusively being homosexual, particularly prior to age 30, when the majority of HPV transmission takes place (Table S4).^7^

Table S4. Distribution of sexual orientation, by gender and age

|  | **Heterosexual** | | **Homosexual** | **Bisexual** | **Heterosexual with homosexual tendencies** | | **Homosexual with heterosexual tendencies** | | **Not sure** | **Total** |
| --- | --- | --- | --- | --- | --- | --- | --- | --- | --- | --- |
| **Females** | |  |  |  |  |  | |  | |  |
| 18-19 | | 84.6% | 0.0% | 7.7% | 6.6% | 0.0% | | 1.1% | | 100.0% |
| 20-24 | | 88.4% | 0.7% | 2.4% | 6.5% | 0.3% | | 1.7% | | 100.0% |
| 25-29 | | 94.7% | 0.6% | 0.8% | 3.4% | 0.0% | | 0.6% | | 100.0% |
| 30-34 | | 94.5% | 0.3% | 0.6% | 3.8% | 0.0% | | 0.8% | | 100.0% |
| 35-39 | | 96.0% | 0.7% | 1.3% | 1.3% | 0.0% | | 0.7% | | 100.0% |
| 40-49 | | 96.1% | 0.5% | 0.5% | 1.8% | 0.0% | | 1.1% | | 100.0% |
| **Males** | |  |  |  |  |  | |  | |  |
| 18-19 | | 86.1% | 2.8% | 0.0% | 9.7% | 0.0% | | 1.4% | | 100.0% |
| 20-24 | | 89.5% | 0.6% | 1.9% | 5.6% | 1.2% | | 1.2% | | 100.0% |
| 25-29 | | 93.1% | 1.5% | 0.5% | 3.9% | 0.0% | | 1.0% | | 100.0% |
| 30-34 | | 96.4% | 1.6% | 0.0% | 1.2% | 0.4% | | 0.4% | | 100.0% |
| 35-39 | | 94.1% | 1.5% | 0.0% | 3.5% | 0.5% | | 0.5% | | 100.0% |
| 40-49 | | 91.7% | 2.3% | 1.1% | 2.5% | 0.6% | | 1.7% | | 100.0% |

**Dynamic model calibration**

The dynamic model calibration has been described previously.^1;2^ Briefly, for Norway, we calibrated 1) type-specific transmission probability of HPV per infected-susceptible partnership, 2) clearance rate of HPV, and 3) progression rate of a high-grade lesion to cancer. For HPV clearance rates we used baseline gender and type-specific regression probabilities from the published literature.^8;9^ To capture the lower transmission probability from male-to-females compared to female-to-males,^4^ we estimated the relative risk (RR) of male-to-female transmission using Hernandez et al 2008;^10^ selecting “any genital” and “cervix/urine” as the sub-sites, and rounding to the nearest tenth. We applied the RR to the female-to-male transmission probability. Female type-specific natural immunity following clearance of first infection was estimated from the calibrated stochastic cervical cancer disease model^11^ and used as an input value in the dynamic model resulting in natural immunity parameters of 0.88 and 0.87 for HPV-16 and -18, respectively. We assumed men do not mount the same level of natural immunity as women^3^ (i.e., male-specific natural immunity of 11% after first infection, for both HPV-16 and -18).

We generated a repository of 100,000 models runs, each using unique parameter values selected from a uniform distribution over the specified ranges. Model outcomes associated with each parameter set were scored according to their fit using a computed composite goodness-of-fit score (summed over the log-likelihood measures of all targets). A cut-off value, equal to the number of targets, was used to determine statistically indistinguishable sets (n=3,160). For the base case analysis, we selected the parameter set that was closest the mean value across all 3,160 “good-fitting” sets (Table S5).

Table S5. Calibrated parameter values

|  | **Variable** | **Baseline probability^a^** | **Search range** | **Mean values from good-fitting sets (n=3,160)** | **Selected for  base case** |
| --- | --- | --- | --- | --- | --- |
| **1. Transmission probability per infected-susceptible partnership** | | | | | |
|  | Female-to-male |  |  |  |  |
|  | HPV-16 | 0.5 | 0.5-1.0 | 0.839 | 0.832 |
|  | HPV-18 | 0.5 | 0.5-1.0 | 0.830 | 0.836 |
|  | Male-to-female |  |  |  |  |
|  | HPV-16 | =Female HPV-16 *0.8 | =Female HPV-16 *0.8 | 0.671 | 0.666 |
|  | HPV-18 | =Female HPV-18 *0.8 | =Female HPV-18 *0.8 | 0.664 | 0.6688 |
| **2. HPV clearance** | |  |  |  |  |
|  | Female^8^ |  |  |  |  |
|  | HPV-16 | 0.2620 | 0-2^b^ | 1.235 | 1.218 |
|  | HPV-18 | 0.4099 | 0-2^b^ | 1.235 | 1.218 |
|  | Male^9^ |  |  |  |  |
|  | HPV-16 | 0.4928 | 0-2^b^ | 1.235 | 1.218 |
|  | HPV-18 | 0.7176 | 0-2^b^ | 1.235 | 1.218 |
| **3. CIN 2, 3 to invasive cancer (HPV-16, -18)** | | **0.002-0.030^c^** | **0-2^b^** | **1.249** | **1.1777** |
|  |  |  |  |  |  |
| HPV: human papillomavirus, CIN: cervical intraepithelial neoplasia. ^a^Baseline probabilities are annual probabilities. ^b^Values represent factors that were multiplied by the baseline probability. ^c^Range represents age-specific values. | | | | | |

**Stochastic Model calibration**

A similar approach as used in the dynamic model was used to calibrate baseline inputs to reflect the natural history of cervical cancer in Norway and has been described previously.^11^ In total, 37 Norwegian-specific calibration targets were defined, which included age-specific prevalence of HPV-16, -18 in women, age-specific prevalence of cervical intraepithelial neoplasia (CIN) 2/3, HPV-16, -18 and other high-risk HPV distributions in high-grade CIN, HPV-16 and -18 distributions in cervical cancer and age-specific cancer incidence. For each calibration target, we determined a point estimate and confidence interval, using population-based sources. For the base case analysis, we used the mean across the 50 “good-fitting” parameter sets.

**Model calibration output**

For the dynamic model, the black bars represent the bounds from the empirical data, grey bars represents model output for five good-fitting sets, and the red diamonds represent the selected parameter set (closest to the mean across all good-fitting sets) used for all analyses. Below is the calibration output for HPV prevalence (Figure S1), cancer incidence (Figure S2) and HPV-type distribution in high-grade precancer and cancer from the stochastic model (Figure S3). For female HPV-16 and -18 prevalence, we used Skjeldestad et al 2008^12^ to informed the bounds for the younger age groups (16-19 years old) and unpublished data (Mari Nygård, MD, PhD, personal communication, Cancer Registry of Norway) to inform the bounds for ages 18-49 years. We assumed study participants to be sexually active and used a weighted average from two sexual behavior studies conducted in Norway^6;7^ to adjust for sexual debut in the younger age groups (57% sexual debut for ages 15-19 years).

Figure S1. Age-specific prevalence of HPV among females from dynamic model


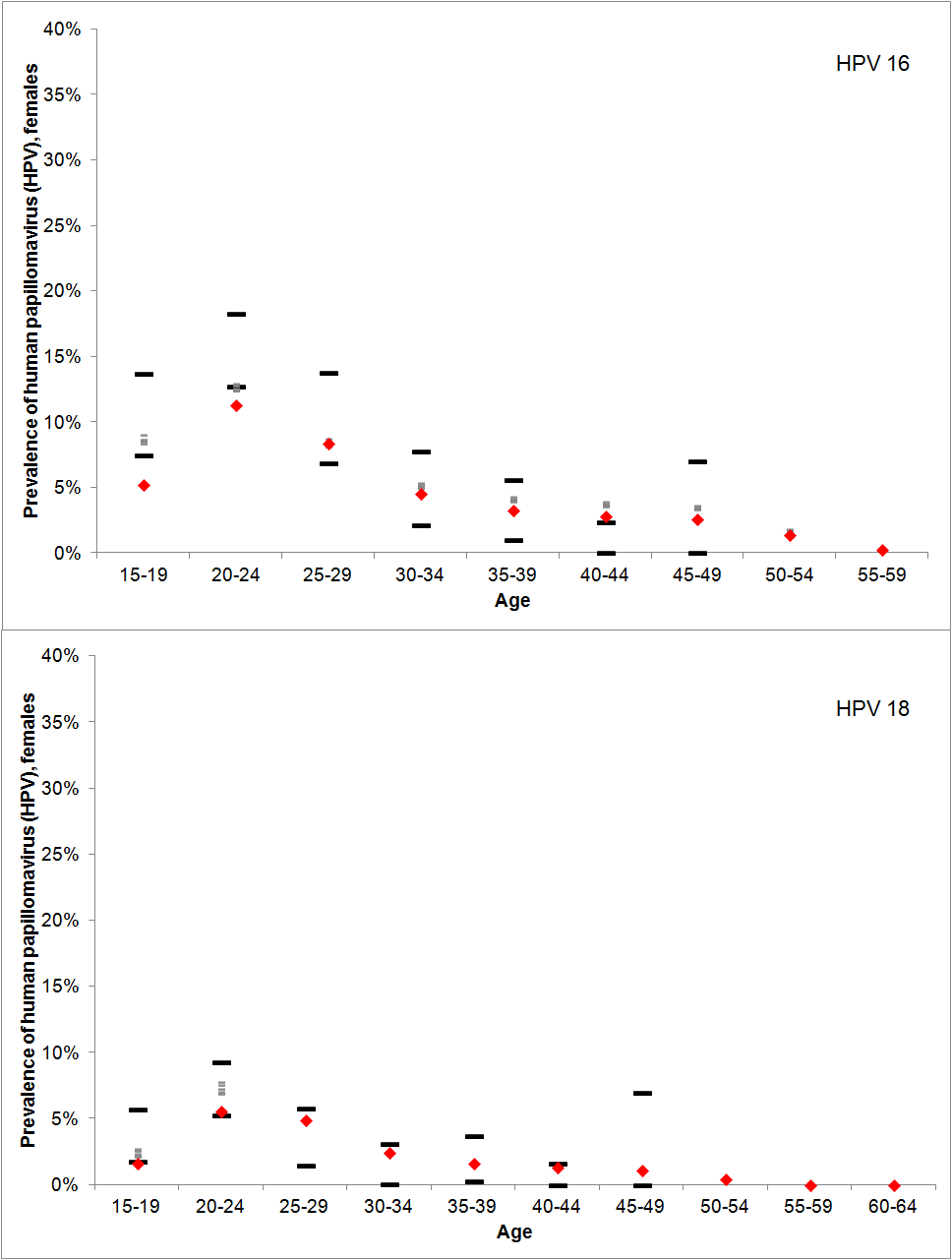


Figure S2. Annual incidence of HPV-16, -18 associated cervical cancer incidence (1953-1969) from the dynamic model


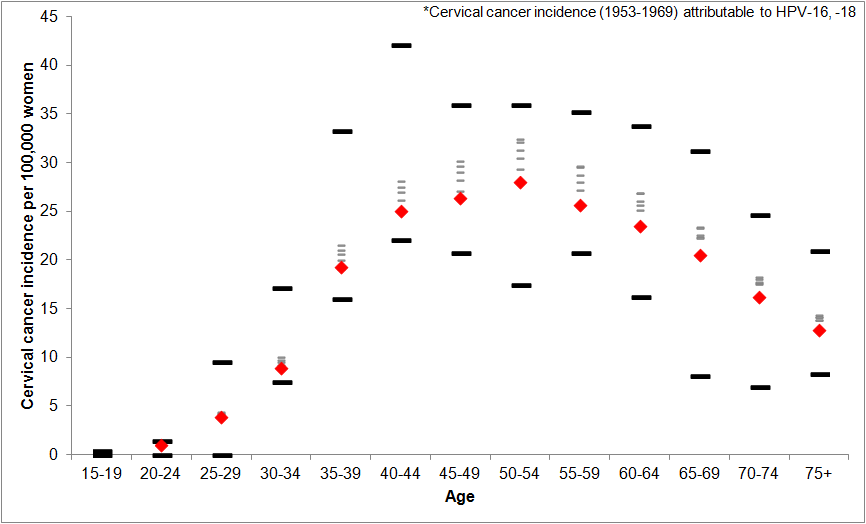


Figure S3. HPV type distribution among females with high-grade precancer (CIN2,3) and invasive cancer from the stochastic cervical cancer model (grey bars represent the top 50 good-fitting parameter sets used in all analyses)


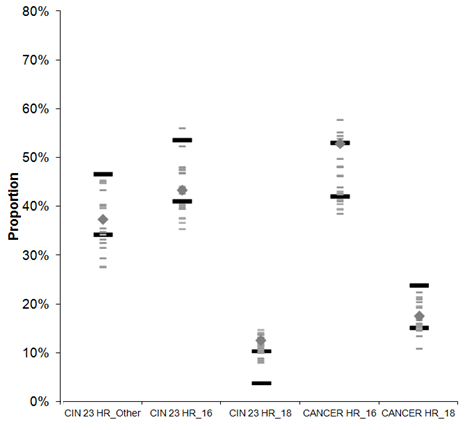


**Inputs for non-cervical diseases**

*Diseases attributable to high-risk HPV-16 and -18*

The dynamic model was used to estimate the gender and type-specific cumulative reductions in HPV-16 and -18 incidences up to age 50, given vaccine efficacy reported in the literature and inclusive of herd immunity benefits. The estimated cumulative reductions were used as inputs into the incidence-based non-cervical cancer models (Figure S4). The incidence of each of the non-cervical cancers, the proportion of cases attributable to vaccine-targeted HPV types (attributable fraction), 5-year survival, disease-specific utility weights, the cost per case (see PART II, below) and excess mortality served as primary inputs to the models. We multiplied the cumulative reductions in HPV from the dynamic model by the attributable fraction for each non-cervical cancer. To project the impact of vaccination, we ran the models both without and with vaccination for each cohort in order to estimate the incremental costs and QALYs associated with the strategies.

**Figure S4.** Model schematic for non-cervical cancers


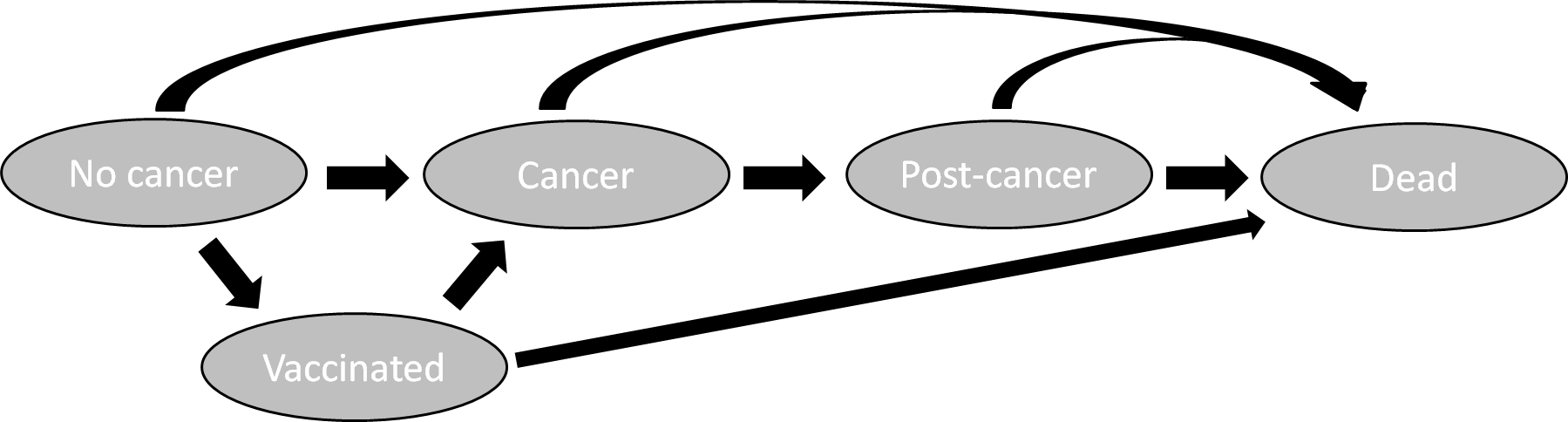


We used data from the Cancer Registry of Norway to inform the expected age-specific cancer incidence in the absence of vaccination and 5-year survival for each oncogenic condition **(main manuscript Table 1)**. The registry is based on a modified version of International Classification of Disease, version 7 or version O (ICD-7/ICD-O).^[[1]](#footnote-1)^ We defined oropharyngeal cancer to include ICD codes C01, C09 and C10 (base of tongue, tonsils and oropharynx). Data specific to Norway (or Northern Europe if Norwegian-specific data was not available) from ICO Data Query System^13^ was used to inform the proportion of cases attributable to vaccine-targeted HPV types (Appendix Table 6). The upper and lower attributable fractions were used in the “optimistic” and “pessimistic” multi-way sensitivity analysis (see main manuscript Methods). It should be noted that the attributable fraction and incidence of non-cervical cancers are prone to misclassification. For example, for anal cancers (ICD-code C21), the topographical "line" separating anal and rectal cancers is unclear. Squamous cell carcinomas coded in rectum may be in fact anal cancers while adenocarcinomas in the anus are most likely rectal cancers. To estimate the proportions in main manuscript Figure 1, HPV-16 and -18 associated cancers were defined as cancers at specific anatomic sites where HPV is commonly found. We applied the attributable fractions of HPV-16 and -18 from main manuscript Table 1 to the average number of new cases per year (2008-2010) from the Cancer Registry of Norway.

*Diseases attributable to low-risk HPV-6 and -11*

To estimate the impact vaccination will have on genital warts, we utilized data on age- and gender-specific incidence of genital warts from two Northern European countries and calibrated the cumulative incidence of having at least one case of genital warts to Norwegian self-reported cumulative risk of 9.5%.^14^ We used Swedish data derived from the national prescription registry to inform younger ages (ages 10-44).^15^ These data included incident cases adjusted to exclude reoccurrences within six month or one calendar year. In the UK, the Health Protection Agency reported the incidence genital warts (new episodes) from England 2002-2011.^16^ We selected the data from 2011 for ages 45-65+, to supplement the data from Sweden. After adjusting the reported genital wart incidence (essentially reducing the age-specific rate) to reflect the cumulative incidence reported in Norway,^14^ the remaining incident cases reported were then assumed to be reoccurrences. For juvenile-onset respiratory papillomatosis (RRP), we specified a model which captured 2011 age-specific fertility rates (Table S1) per woman, obtained from Statistics Norway, and applied the Norwegian incidence rate of RRP^17^ per live birth. The proportion of RRP attributable to low-risk HPV-6 and -11 was assumed to be 100% (Table S6) but varied in sensitivity analysis (80-100%). The impact of vaccination herd immunity benefits on HPV-6 and -11 warts and RRP was assumed to be proportional to that of HPV-18 estimated from the dynamic model. We estimated the monetary costs associated with treating genital warts or RRP using Norwegian-specific guidelines (See **PART II**, below).

Table S6. Summary of attributable fraction of vaccine-targeted HPV in non-cervical disease

|  | **HPV 16** | **HPV 18** | **HPV 6/11** | **Setting** |
| --- | --- | --- | --- | --- |
| Vaginal, mean (CI) | 63% (45-79) | 3% (0-15) | nr | Northern Europe^13^ |
| Vulva, mean (CI) | 38% (32-44) | 6% (3-10) | nr | Northern Europe^13^ |
| Penile, mean (CI) | 42% (23-63) | 4% (0-20) | nr | Northern Europe^13^ |
| Anal, mean (CI) | 73% (70-77) | 9% (7-12) | nr | Northern Europe^13^ |
| Oropharyngeal, mean (CI) | 53% (41-63) | 1% (0-6) | nr | Norway^18^ |
| JoRRP | nr | nr | 100% (80-100) | Multiple^19^ |
| Warts | nr | nr | 90% (80-100) | Multiple^19^ |
| CI: 95% confidence interval, HPV: Human papillomavirus, JoRRP: Juvenile-onset recurrent respiratory papillomatosis, nr: not relevant. | | | | |

**Screening assumptions**

All vaccination scenarios assumed screen-eligible women followed the current cervical cancer screening algorithm according to published compliance rates (i.e., status quo)^20^ and costs^11^ (Table S7). Status quo screening involved primary cytology (Pap) testing every three years. Women with a high-grade result are referred directly to colposcopy with biopsy and treated if a high-grade lesion is confirmed. Women with atypical cells or a low-grade result are referred to combination testing (HPV with cytology) six months after primary index test. Individuals who are both HPV-positive and cytology-positive (i.e., atypical cells or worse) are referred to colposcopy with biopsy, while women with an HPV-negative and mild cytology result are returned to their normal screening schedule. Persistently HPV-positive and cytology-negative women are referred to colposcopy biopsy while, if at any point, a woman returns an HPV-positive, cytology-positive result, she will be referred directly to colposcopy/biopsy. We assumed that 65% of women attend screening every three years, 25% attend less frequently than recommended (i.e., every 4-8 years) and 10% never attend. We also assumed compliance after an abnormal result was imperfect, such that 35% do not comply within one year (but are not assumed to be permanently lost to follow-up).

Table S7. Screening cost inputs

|  | **Total^a^** |
| --- | --- |
| Conventional cytology | $49 |
| HPV DNA testing | $62 |
| Office visit, time & transport | $160 |
| Colposcopy with biopsy | $337 |
| High-grade precancer | $2,160 |

**PART II:** COSTS

**HPV vaccine**

In 2011, a pharmaceutical statistics company (Farmastat^21^), reported that 34,313,964 Norwegian Kroner (NOK) was spent on 76,220 doses of the HPV vaccine (098732 Gardasil INJ 10X0.50MLSPR), resulting in a price of approximately NOK 450 per dose (≈$75). This amount is exclusive of value added tax (VAT). Estimates from 2012 show a stable cost per dose. To estimate administration costs, we assumed nurses travel between schools, use approximately 15-20 minutes per student (≈37.5 students per day) and 60 minutes of travel per day (personal communication). In Norway, non-specialty nurses earn approximately NOK33,600 per month^5^ and approximately NOK 564,480 per year (including 40% fringe costs). These estimates imply administration and supplies to be NOK 84 (≈$13.83) per dose. This is in line with school-based administration estimates reported in the UK.^22^

**Treatment cost estimates for non-cervical HPV-related conditions**

Norwegian-specific treatment costs of non-cervical HPV-related diseases have not been previously published; therefore, we estimated the direct medical costs for the diagnosis, treatment and 5-year surveillance (discounted 4% per year) using a combination of primary data from the National Patient Registry, national guidelines and clinical judgment. All cost estimates used in the current analysis are shown in Table S8, while we present more detailed estimation and rationales below using anal cancer and genital warts to exemplify our process. Additional costing information may be obtained from the authors upon request. To estimate costs associated treatment, we based costs on official national hospital-based DRG reimbursement tariffs^23^ and physician fee schedules^24^ using official treatment guidelines, where available.^25^ For each HPV condition we sought expert opinion from Norwegian oncologists and clinicians to assist in quantifying resource use and outline the typical patient care pathway. In addition, data from the 2011 National Patient Registry provided additional insight on DRG utilization for each cancer specified by ICD codes. The diagnosis of all stages consisted of an initial physician office visit, site-specific assessment, biopsy and examination of the biopsy for pathology. Follow-up care for post treatment surveillance was cancer-specific, but generally assumed four follow-up visits the first year, followed by biannual and annual visits for year two and years 3-5, respectively. Data from the Cancer Registry of Norway^26^ informed the distribution of stage at detection to give the stage-weighted average lifetime cost per case. The proportion of direct non-medical costs (transport) and patient time for all non-cervical conditions (≈15%) was estimated from detailed cervical cancer costing and applied to baseline direct medical costs for all other non-cervical HPV-related conditions. Influential inputs were varied for each condition and we determined that uniformly applying +/- 25% would generate reasonable upper and lower bounds for our cost estimates. The upper and lower bounds for Norwegian direct medical treatment costs were either inclusive or slightly lower than costing point estimates recently reported for HPV-related cancers in Denmark (a 3-year hospital sector perspective exclusive of costs in the year before diagnosis).^27^ Monetary costs were measured in 2010 NOK and converted to US dollars (US $) using the average annual 2010 exchange rate (US $1=NOK6.05).^28^

Table S8. Summary of treatment costs for HPV-related diseases, direct medical and total costs

|  | **Direct medical** | **Total^a^** | **LB^a^** | **UB^a^** |
| --- | --- | --- | --- | --- |
| Cervix |  |  |  |  |
| High-grade precancer |  | $2,160 |  |  |
| Local | $23,500 | $25,800 | $19,300 | $32,200 |
| Regional | $41,100 | $51,600 | $38,700 | $64,500 |
| Distant | $48,800 | $59,600 | $44,700 | $74,500 |
| Vagina | $22,900 | $26,400 | $19,800 | $33,000 |
| Vulva | $24,200 | $27,900 | $20,900 | $34,800 |
| Penis | $15,200 | $17,500 | $13,100 | $21,800 |
| Anus | $32,600 | $37,500 | $28,100 | $46,900 |
| Oropharynx | $42,600 | $49,000 | $36,800 | $61,300 |
| JoRRP | $116,400 | $133,800 | $100,400 | $167,300 |
| Genital warts | $350 | $400 | $300 | $500 |
| ^a^Incusive of direct and nondirect medical costs. Assumes an average of 15% (estimated from the weighted average of cervical cancer treatment) additional costs due to nondirect medical costs (such as transportation and patient time) for all non-cervical diseases. Lower bounds (LB) and upper bounds (UB) estimated by taking 75% and 125% of the total cost estimate. ($1USD= NOK6.05, 2010) | | | | |

*Example 1: Anal cancer treatment:*

In developing stage-specific estimates for the average lifetime cost of anal cancer, treatment strategies were based on new treatment recommendations set forth by the Norwegian Gastrointestinal Cancer Group (NGICG) and KVIST,^29^ and expert opinion from Oslo University Hospital, Ullevål (personal communication: Marianne G. Guren, MD, PhD). Treatment costs of stage T1/T2 (N0) cancer consisted of the costs related to CT/MRI for dose planning, inpatient care (consisting of five radiotherapy fractions plus one course of MiFu chemotherapy), and outpatient care (consisting of 22 fractions of radiotherapy (total of 54GY=27 fr)). Treatment costs related to T3/T4 (N0/N+) consisted of 58GY (29 fractions) plus two courses of MiFu. For T3/T4 (N0/N+) cancer we assumed two separate inpatient stays for which patients received chemotherapy and ten fractions of radiotherapy. The remaining fractions were delivered on an outpatient basis. We also assumed 18% of patients were readmitted for toxicity and 13% had residual tumor and were treated surgically with abdominoperineal resection.^30^ For all stages we applied the cost of weekly consultations throughout the course of primary treatment. We estimated the direct medical average cost per case to be NOK196,000 or approximately $32,600. Our cost estimates varied from $30,800-34,100 (NOK186,000-206,000) when we assumed different baseline inputs (Figure S5). Finally, to ensure sensitivity analysis captured all plausible ranges, we varied the base case estimates by +/-25%, resulting in $24,300-40,500 (NOK147,000-245,000). To capture the direct non-medical costs such as transportation to/from appointments and patient time, 15% was added to baseline calculations resulting in a total direct medical and nonmedical cost of $37,500 (NOK226,800). The lower and upper bounds, used in the “optimistic” and “pessimistic” scenario analysis, equated to $28,100 and 46,900, respectively (NOK170,100-283,500)).

Figure S5. Impact of influential parameters on the average direct medical cost of anal cancer


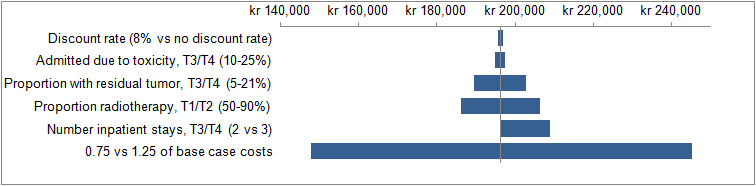


*Example 2: Genital warts*

Treatment algorithms reflecting local practice were elicited from experts at Oslo University Hospital, Olafia Clinic (personal communication: Harald Moi, MD, PhD and Anne Olsen, MD, PhD) and used in combination with recently published European guidelines^31^ to generate estimates of the cost of an average case of genital warts. We assumed 25% reoccurred after three months, 13% of the reoccurrences persisted in year two and 2% continued to persist into year three. For the first case of warts, we assumed that 90% are treated with a solution or cream (Podophyllotoxin 5g and to a lesser extent Imiquimod 5%), 8% received cryotherapy plus Podophyllotoxin 5g, and 2% received laser treatment as first line therapy. For first time reoccurrences, 40% received solution or cream (higher proportion receiving Imiquimod 5%), 30% received combination cryotherapy plus Podophyllotoxin 5g and 30% received laser treatment. For the few cases persisting into year three, 20% received solution/cream (nearly all receive Imiquimod 5%), 30% received cryotherapy plus Podophyllotoxin, and 50% received laser therapy. We estimated that an average case of genital warts cost approximately $350 (NOK 2099) (Figure S6). After including 15% (for nondirect medical costs and patient time), the total costs associated with an average case of genital warts came to $400 (NOK2410) with a lower bound of $300 (NOK1810) and an upper bound of $500 (NOK3010).

Figure S6. Impact of influential parameters on the average direct medical cost of an episode of genital warts


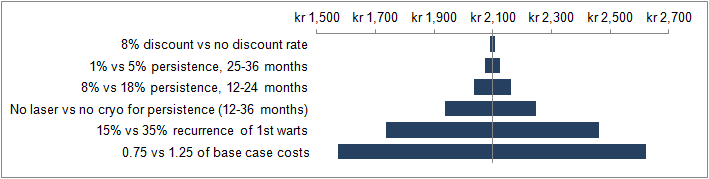


**Part III:** Additional results

Table S9 shows the estimated annual number of cases of HPV-related cancers under base case assumptions for vaccine properties (see text of main manuscript).

Table S9. Projected annual number of cases for various vaccination scenarios

|  | **Observed number of cases, average 2008-2010** | **Projected annual number of cases**^a^ | | | | **Difference** | | |
| --- | --- | --- | --- | --- | --- | --- | --- | --- |
|  | **No vaccination** | **Girls-only vaccination (71%)** | **Girls + boys vaccination (71%)** | **Girls-only vaccination (90%)** | **Girls-only vaccination (90%), no additional herd immunity for boys** | **Girls + boys vaccination (71%) vs. girls-only (71%)** | **Girls-only vaccination (90%) vs. girls + boys (71%)** | **Girls-only vaccination (90%), no additional herd immunity for boys vs. girls + boys (71%)** |
| Cervix^b^ | 306 | 179 | 162 | 135 | 135 | 17 | 27 | 27 |
| Vulva | 82 | 53 | 49 | 48 | 48 | 3 | 2 | 2 |
| Vagina | 14 | 7 | 6 | 5 | 5 | 1 | 0 | 0 |
| Anus, female | 47 | 16 | 12 | 10 | 10 | 3 | 2 | 2 |
| Oropharynx, female | 37 | 21 | 19 | 18 | 18 | 2 | 1 | 1 |
| Penis | 49 | 35 | 28 | 29 | 35 | 6 | -1 | -6 |
| Anus, male | 23 | 11 | 6 | 7 | 11 | 5 | -1 | -5 |
| Oropharynx, male | 91 | 61 | 48 | 50 | 61 | 13 | -2 | -13 |
| **Total, both genders** | **648** | **381** | **331** | **302** | **323** | **51** | **28** | **8** |
| Total, female | 486 | 275 | 249 | 217 | 217 | 26 | 32 | 32 |
| Total, male | 162 | 106 | 82 | 85 | 106 | 25 | -4 | -25 |
| ^a^Projections reflect the expected number of cases per year using expected cancer reductions for the last cohort included in this analysis. The reduction in non-cervical HPV related cancers due to vaccination are assumed to be proportional to the reduction in cumulative risk of acquiring HPV prior to age 50 and multiplied by the disease-specific HPV-16, -18 attributable fractions. ^b^Projected reduction in risk of cervical cancer is estimated from the stochastic disease model and in the context of current cervical cancer screening compliance. | | | | | | | | |

Table S10 shows the discounted incremental cost and QALY for each HPV-related cancer associated with the base case analysis.

**Table S10.** Discounted incremental costs and QALYs

|  | No vaccine to girls only (71%) | | Girls only (71%) to girls + boys (71%) | |
| --- | --- | --- | --- | --- |
|  | Disc. incremental cost | Disc. incremental QALYs | Disc. incremental cost | Disc. incremental QALYs |
| Vulva | -$72 | 0.01947 | -$6 | 0.00163 |
| Vaginal | -$16 | 0.00324 | -$1 | 0.00027 |
| Anal, female | -$116 | 0.02706 | -$10 | 0.00226 |
| Oropharyngeal, female | -$80 | 0.01268 | -$7 | 0.00106 |
| Cervical cancer | -$864 | 0.13902 | -$149 | 0.02463 |
| JoRRP | -$3 | 0.00002 | $0 | 0.00000 |
| Warts, females | -$453 | 0.03015 | -$40 | 0.00267 |
| Penile | -$21 | 0.02805 | -$11 | 0.00152 |
| Anal, male | -$37 | 0.00565 | -$20 | 0.00280 |
| Oropharyngeal, male | -$138 | 0.03338 | -$76 | 0.00812 |
| Warts, males | -$334 | 0.02177 | -$144 | 0.00945 |
| Vaccine ($75/dose) | $3,733 |  | $3,733 |  |
| Total | $1,600 | 0.32048 | $3,269 | 0.05440 |
| ICER | $4,992 | | $60,085 | |
| Disc.: Discounted (4% per year), ICER: incremental cost-effectiveness ratio, JoRRP: Juvenile-onset recurrent respiratory papillomatosis, QALY: Quality-adjusted life year | | | | |

References

(1) Kim JJ, Goldie SJ. (2009) Cost effectiveness analysis of including boys in a human papillomavirus vaccination programme in the United States. *BMJ* 339.

(2) Kim JJ, Goldie SJ. (2008) Health and economic implications of HPV vaccination in the United States. *New England Journal of Medicine* 359: 821-832.

(3) Lu B, Viscidi RP, Wu Y, Lee JH, Nyitray AG, Villa LL et al. (2012) Prevalent Serum Antibody Is Not a Marker of Immune Protection against Acquisition of Oncogenic HPV16 in Men. *Cancer Research* 72: 676-685.

(4) Moscicki AB, Schiffman M, Burchell A, Albero G, Giuliano AR, Goodman MT et al. (2012) Updating the Natural History of Human Papillomavirus and Anogenital Cancers. *Vaccine* 30: F24-F33.

(5) Statistics Norway. Available: http://www.ssb.no/english/. Accessed 10 January 2012

(6) Pedersen W, Samuelsen SW. (2003) [New patterns of sexual behavior among adolescents]. *Journal of the Norwegian Medical Society (Tidsskrift for Den norske legeforening)* 21: 3006-3009.

(7) Træen B, Stigum H, Magnus P. (2003) [Report from the sexual behavior survey in 1987, 1992, 1997, 2002]. Norwegian Institute for Public Health, Division of Epidemiology.

(8) Insinga RP, Perez G, Wheeler CM, Koutsky LA, Garland SM, Leodolter S et al. (2011) Incident Cervical HPV Infections in Young Women: Transition Probabilities for CIN and Infection Clearance. *Cancer Epidemiology Biomarkers & Prevention* 20: 287-296.

(9) Giuliano AR, Lee JH, Fulp W, Villa LL, Lazcano E, Papenfuss MR et al. (2011) Incidence and clearance of genital human papillomavirus infection in men (HIM): a cohort study. *Lancet* 377: 932-940.

(10) Hernandez BY, Wilkens LR, Zhu X, Thompson P, McDuffie K, Shvetsov YB et al. (2008) Transmission of human papillomavirus in heterosexual couples. *Emerging Infectious Diseases* 14: 888-894.

(11) Burger EA, Ortendahl JD, Sy S, Kristiansen IS, Kim JJ. (2012) Cost-effectiveness of cervical cancer screening with primary human papillomavirus testing in Norway. *Br J Cancer* 106: 1571-1578.

(12) Skjeldestad FE, Mehta V, Sings HL, Ovreness T, Turpin J, Su L et al. (2008) Seroprevalence and genital DNA prevalence of HPV types 6, 11, 16 and 18 in a cohort of young Norwegian women: study design and cohort characteristics. *Acta Obstetricia et Gynecologica Scandinavica* 87: 81-88.

(13) World Health Organization. ICO (Institut Català d'Oncologia). Information Centre on Human Papilloma Virus (HPV) and Cervical Cancer: Data Query System. Available: http://www.who.int/hpvcentre/en/. Accessed 15 November 2012.

(14) Kjaer SK, Tran TN, Sparen P, Tryggvadottir L, Munk C, Dasbach E et al. (2007) The burden of genital warts: a study of nearly 70,000 women from the general female population in the 4 Nordic countries. *Journal of Infectious Diseases* 196: 1447-1454.

(15) Leval A, Herweijer E, Arnheim-Dahlstrom L, Walum H, Frans E, Sparen P et al. (2012) Incidence of Genital Warts in Sweden Before and After Quadrivalent Human Papillomavirus Vaccine Availability. *Journal of Infectious Diseases* 206: 860-866.

(16) Health Protection Agency. Number & rates of anogenital warts diagnosed in England, 2002-2011.Available: www.hpa.org.uk/webc/HPAwebFile/HPAweb_C/1296688631209. Accessed 11 January 2013.

(17) Omland T, Akre H, Vardal M, Brondbo K. (2012) Epidemiological aspects of recurrent respiratory papillomatosis: A population-based study. *Laryngoscope* 122: 1595-1599.

(18) Mork J, Møller Br, Dahl T, Bray F. (2010) Time trends in pharyngeal cancer incidence in Norway 1981−2005: a subsite analysis based on a reabstraction and recoding of registered cases. *Cancer Causes and Control* 21: 1397-1405.

(19) Lacey CJN, Lowndes CM, Shah KV. (2006) Burden and management of non-cancerous HPV-related conditions: HPV-6/11 disease. *Vaccine* 24: 35-41.

(20) Cancer Registry of Norway. (2012) [2009 Annual Report Population-based Screening against Cervical Cancer]. Available: http://www.kreftregisteret.no/

(21) Farmastat AS. 2013. http://www.farmastat.no.

(22) Jit M, Chapman R, Hughes O, Choi YH. (2011) Comparing bivalent and quadrivalent human papillomavirus vaccines: economic evaluation based on transmission model. *British Medical Journal* 343.

(23) Norwegian Directorate of Health. [Activity-based funding 2010/2011]. Available: http://www.helsedirektoratet.no/finansieringsordninger/regelverk_innsatsstyrt_finansiering__isf__2011_78057. Accessed 10 December 2010.

(24) Norwegian Medical Association. [Normal tariff for private general practice 2010-2011]. Available: http://www.legeforeningen.no/normaltariff/Fastlegetariff_2010.pdf. Accessed 1 November 2010.

(25) Oncolex: Encyclopedia for Diagnosing and Treating Cancer. http://www.oncolex.no.

(26) Cancer Registry of Norway: Institute of population-based cancer research. http://www.kreftregisteret.no.

(27) Olsen J, Jorgensen TR, Kofoed K, Larsen HK. (2012) Incidence and cost of anal, penile, vaginal and vulvar cancer in Denmark. *Bmc Public Health* 12.

(28) Federal Reserve. Historical Rates for the Norwegian Krone. Available: http://www.federalreserve.gov/RELEASES/H10/Hist/dat00_no.htm. Accessed 13 June 2011.

(29) NGICG and KVIST. [Professional recommendations for treatment of anal cancer]. Available: http://ngicg.no/ngicg/handlingsprogram/faglige_anbefalinger/content/filelist_d92e9f7e-226a-4d54-8c2d-bc4ea29fbfb9/1348047422069/faglige_anbefalinger_for_behandling_ved_analcancer_21211.pdf. Accessed 30 June 2012.

(30) Bentzen AG, Guren MG, Wanderas EH, Frykholm G, Tveit KM, Wilsgaard T et al. (2012) Chemoradiotherapy of Anal Carcinoma: Survival and Recurrence in an Unselected National Cohort. *International Journal of Radiation Oncology Biology Physics* 83: E173-E180.

(31) Lacey CJN, Woodhall SC, Wikstrom A, Ross J. (2013) 2012 European guideline for the management of anogenital warts. *Journal of the European Academy of Dermatology and Venereology* 27: e263-e270.

1. This study uses data from the Cancer Registry of Norway. Interpretation and reporting of these data are the sole responsibility of the authors and has not been subject to acceptance from the Cancer Registry. [↑](#footnote-ref-1)
